# Wilson disease in Costa Rica: Pediatric phenotype and genotype characterization

**DOI:** 10.1002/jmd2.12098

**Published:** 2020-02-06

**Authors:** Monica Penon‐Portmann, Stephanie Lotz‐Esquivel, Alejandra Chavez Carrera, Mildred Jiménez‐Hernández, Danny Alvarado‐Romero, Sharon Segura‐Cordero, Fiorella Rimolo‐Donadio, Francisco Hevia‐Urrutia, Alfredo Mora‐Guevara, Manuel Saborío‐Rocafort, Gabriela Jiménez‐Arguedas, Ramsés Badilla‐Porras

**Affiliations:** ^1^ Servicio de Genética y Enfermedades Metabólicas, Departamento de Pediatría, Hospital Nacional de Niños, “Dr. Carlos Sáenz Herrera”, Caja Costarricense de Seguro Social & Sistema de Estudios de Posgrado Universidad de Costa Rica San José Costa Rica; ^2^ Division of Medical Genetics, Department of Pediatrics & Institute for Human Genetics University of California San Francisco San Francisco California; ^3^ Clínica Multidisciplinaria de Enfermedades Raras y Huérfanas & Unidad de Investigación, Departamento de Medicina Interna, Hospital San Juan de Dios Caja Costarricense de Seguro Social San José Costa Rica; ^4^ Escuela de Medicina, Colegio de Ciencias de la Salud Universidad San Francisco de Quito Quito Ecuador; ^5^ Programa Nacional de Tamizaje Neonatal Caja Costarricense de Seguro Social San José Costa Rica; ^6^ Laboratorio Nacional de Tamizaje Neonatal y Alto Riesgo Caja Costarricense de Seguro Social San José Costa Rica; ^7^ Unidad de Trasplante, Departamento de Cirugía, Hospital Nacional de Niños, “Dr. Carlos Sáenz Herrera” Caja Costarricense de Seguro Social San José Costa Rica; ^8^ Servicio de Gastroenterología, Sección de Medicina, Hospital San Juan de Dios Caja Costarricense de Seguro Social San José Costa Rica; ^9^ Servicio de Gastroenterología, Departamento de Pediatría, Hospital Nacional de Niños, “Dr. Carlos Sáenz Herrera” Caja Costarricense de Seguro Social San José Costa Rica

**Keywords:** acute liver failure, *ATP7B*, genotype and phenotype, pediatric, Wilson disease

## Abstract

**Introduction:**

The prevalence of Wilson disease (WD) in Costa Rica is among the highest reported in the world, 4.9:100 000. Previous investigators have also described a burden of autosomal recessive conditions in this country. Genetic testing for WD began in 2010 as a strategy for earlier detection due to the country's high prevalence. Here we describe what we have learned about the genotype and phenotype of the Costa Rican pediatric population with WD.

**Methods:**

We completed a retrospective review of medical records from pediatric individuals (<18 years of age) with molecular testing for *ATP7B* between 2010 and 2015. We documented phenotype and genotype for cases with WD as defined by the international scoring system.

**Results:**

Thirty‐four WD cases from 28 families were included, 15 female and 19 male patients. The most frequent pathogenic variant in *ATP7B* was NM_000053:c.3809A>G, p.Asn1270Ser, with 58.8% of affected individuals homozygous for this variant. Age of diagnosis ranged from 1 to 17 years, with an average of 8.8 ± 3.6 years. All individuals who presented with acute liver failure (n = 6) were homozygous for the p.Asn1270Ser variant (Chi‐squared, *P* < .05).

**Discussion:**

Molecular testing has facilitated the detection of presymptomatic patients with WD in Costa Rica. We hope that ongoing efforts in the delivery of clinical services lead to optimized molecular screening for WD and other genetic conditions in Costa Rica.

**SYNOPSIS**

Here we describe what we have learned about the genotype and phenotype of the Costa Rican pediatric population with WD.

## 1 INTRODUCTION

Wilson disease (WD) is an autosomal recessive disorder in which copper's metabolism is altered due to pathogenic variants in the *ATP7B* gene. The condition's phenotype can vary widely from acute liver failure (ALF) to neuropsychiatric manifestations, and occurs as a result of the toxic effects of excess copper in tissues.^1^, ^2^, ^3^ WD is a treatable condition, and early detection through molecular testing can decrease morbidity and mortality.^4^, ^5^ Costa Rica has the highest prevalence of WD in the world: 4.9:100 000, as per previous reports, as well as a burden of other autosomal recessive conditions.^6^, ^7^ Previous studies have reported a high rate of acute liver failure among WD patients in Costa Rica, approximately 17% in children, in comparison to other reports where only 5% was observed.^8^, ^9^, ^10^, ^11^ To address this, the Medical Genetics and Metabolism Department at the National Children's Hospital (HNN) now provides molecular diagnoses for a group of conditions including Wilson disease (OMIM #277900). Molecular testing for early detection was implemented as a strategic measure to reduce morbidity and mortality.^12^, ^13^, ^14^ The project's goal was to optimize the benefits of molecular testing through extended familial cascade screening, genetic counseling, and reproductive planning.^15^

In this study, we characterize the phenotype and genotype of the Costa Rican pediatric population with WD who had molecular testing between 2010 and 2015 and compare the results with a previous report from our center to demonstrate the impact of strategic measures in the delivery of genetics services.

## 2 METHODS

We completed a retrospective review of medical records from pediatric individuals (<18 years of age) with molecular testing for *ATP7B* between 2010 and 2015 (n = 140). We included patients with WD defined as a score ≥ 4 points in the Leipzig or EASL scoring system (n = 34).^5^

### 2.1 Phenotype

Phenotypic data was collected from medical records. Phenotype was defined by clinical presentation at time of diagnosis as: presymptomatic, hepatic, acute liver failure (ALF), neuropsychiatric, or coombs‐negative acute hemolytic anaemia.^5^ ALF was defined as onset of clinical symptoms <8 weeks with evidence of impaired hepatic function, INR > 2, and any degree of encephalopathy.^16^

### 2.2 Genotype

Molecular testing for *ATP7B* began in 2010 with DNA PCR amplification and restriction enzyme fragment length polymorphism testing.^17^ An initial 4‐exon panel included exons 6, 7, 8, and 18, with variants p.Met645Arg, p.Met665Ile, p.Leu708Pro, and p.Asn1270Ser (GRCh37/hg19, NM_000053). This strategy was later expanded to a 7‐exon panel that included exons 6, 7, 8, 14, 17, 18, and 21. This selection was based on previous Costa Rican population estimates on variant frequencies.^17^ By 2013, the HNN testing center had the ability to sequence by the Sanger method all 21 exons and splicing regions in *ATP7B*.

### 2.3 Statistical analysis

We transferred the information gathered into a collection sheet using the Epi Info Software, where we compiled Leipzig scores to determine patient inclusion.^5^ Statistical analyses were performed using the STATA version 14 and R Software version 3.4.4.

## 3 RESULTS

A total of 140 pediatric individuals had molecular testing for *ATP7B* variants between 2010 and 2015, due to liver disease of unknown etiology or suspicion of WD due to family history. A total of 34 pediatric patients, from 28 families, were confirmed to have WD by a score ≥ 4 points in the Leipzig scoring system. From the 34 diagnosed patients, 23 were new and 11 had been previously reported.^18^, ^19^ Previously reported patients had prior clinical diagnosis and were later confirmed molecularly (Table 1). There were 15 female and 19 male patients diagnosed with no significant age difference between sexes (females = 7.9 ± 4.5 years, males = 9.4 ± 2.6 years, Welch ANOVA *P* = .2654). Age of diagnosis ranged from 1‐17 years, with an average of 8.8 ± 3.6 years. Those presenting with symptoms upon initial evaluation had an average age of 10.1 ± 2.1 years.

**Table 1:** Characteristics of pediatric patients with WD in Costa Rica at presentation (n = 34)

Family‐case	Province	Age (years)	Sex	Kinship	Genotype	Phenotype	Previously reported	Leipzig score	KF ring	24‐hour urinary copper (μmol/24 hours)	Ceruloplasmin (g/L)/ceruloplasmin ferroxidase activity (IU/L)	Hemolytic anemia coombs‐neg	INR	AST/ALT (IU/L)	Total bilirubin (mg/dL)	Liver copper (μg/g dry wt)	Modified Nazer score (if ALF)
1‐1*	San José	6.4	Female	Sibling	p.N1270S/p.N1270S	Hepatic	+	8	–	14.25	0.04 g/L	–	1.00	38/62	0.7	–	–
1‐2	San José	10.3	Female	Sibling	p.N1270S/p.N1270S	Presymptomatic	+	8	–	6.9	1 IU/L	–	1.00	85/121	0.6	–	–
2‐1*	Alajuela	8.1	Male	–	p.N1270S/p.N1270S	Hepatic	–	8	–	1.41	0.00 g/L	–	1.00	184/406	0.4	11.2	–
3‐1*	Puntarenas	7.5	Male	Sibling	p.N1270S/p.N1270S	Hepatic	+	8	–	4.8	7 IU/L	–	1.00	193/341	0.5	–	–
3‐2	Puntarenas	4.5	Male	Sibling	p.N1270S/p.N1270S	Presymptomatic	+	8	–	5.12	13 IU/L	–	–	127/174	0.8	–	–
4‐1*	San José	11.1	Male	Sibling	p.N1270S/p.N1270S	Acute liver failure	–	5	–	–	–	+	2.64	263/45	12.4	–	10
4‐2	San José	2.6	Female	Sibling	p.N1270S/p.N1270S	Presymptomatic	–	6	–	0.6	0.00 g/L	–	1.00	47/35	0.4	–	–
5‐1*	Limón	12.4	Male	–	p.N1270S/p.L708P	Hepatic	–	9	+	3	0.00 g/L	–	1.40	98/30	1.0	–	–
6‐1*	Heredia	9.5	Male	Sibling	p.N1270S/p.L708P	Hepatic	–	11	–	22.96	0.09 g/L	+	1.46	216/123	12.1	4.66	–
6‐2	Heredia	15.7	Male	Sibling	p.N1270S/p.L708P	Presymptomatic	–	8	–	1.19	0.03 g/L	–	–	–	–	4.75	–
7‐1*	Puntarenas	12.1	Female	–	p.N1270S/p.N1270S	Acute liver failure	–	13	–	43.39	0.00 g/L	+	3.15	236/101	30.1	–	11
8‐1*	San José	8.3	Female	–	p.N1270S/p.N1270S	Hepatic	–	9	–	10	0.04 g/L	–	1.77	102/60	0.9	2.1	–
9‐1*±	San José	9.8	Female	–	p.N1270S/p.N1270S	Acute liver failure	–	13	–	26.45	0.00 g/L	+	2.90	161/22	46.7	–	10
10‐1*±	Cartago	13.7	Female	3rd Cousin	p.N1270S/p.N1270S	Acute liver failure	–	15	+	162.14	9 IU/L	+	3.23	127/19	47.7	–	9
10‐2	San José	17.9	Female	3rd Cousin	p.N1270S/p.T1434M	Presymptomatic	–	9	–	16.1	0.17 g/L	–	1.06	22/16	0.8	14.8	–
11‐1	San José	4.6	Female	–	p.N1270S/p.N1270S	Presymptomatic	+	6	–	1.07	6 IU/L	–	1.10	52/56	0.2	–	–
12‐1	San José	13.1	Female	–	p.N1270S/p.M645R	Presymptomatic	–	8	–	17.6	0.00 g/L	–	1.04	237/104	0.4	–	–
13‐1*	San José	11.8	Male	–	p.N1270S/p.N1270S	Coombsneg hemolytic anemia	+	8	–	2.5	11 IU/L	+	1.40	124/16	8.1	–	–
14‐1*†	Cartago	9.8	Male	–	p.N1270S/p.N1270S	Acute liver failure	–	10	–	–	0.10 g/L	+	2.46	147/21	35.9	–	9
15‐1	Guanacaste	6.3	Male	–	p.N1270S/–	Presymptomatic	+	4	–	1.59	35 IU/L	–	1.00	127/206	0.6	25.3	–
16‐1	San José	1.7	Female	–	p.N1270S/p.L708P	Presymptomatic	+	8	–	6.8	0.00 g/L	–	1.10	43/25	0.4	–	–
17‐1	San José	10.1	Male	–	p.N1270S/p.N1270S	Presymptomatic	–	10	–	25.5	0.03 g/L	–	1.15	144/186	0.8	6.8	–
18‐1*	Puntarenas	5.3	Female	Sibling	p.M645R/–	Presymptomatic	–	7	–	11.3	0.00 g/L	–	1.06	87/111	0.4	23.77	–
18‐2	Puntarenas	5.9	Female	Sibling	p.M645R/–	Presymptomatic	–	5	–	7.84	0.00 g/L	–	–	–	–	–	–
19‐1*	San José	8.1	Female	–	p.N1270S/p.N1270S	Acute liver failure	–	13	+	71.65	6 IU/L	+	3.15	219/36	50.8	5.59	11
20‐1*	San José	10.5	Male	–	p.N1270S/–	Hepatic	+	9	+	7.16	11 IU/L	–	1.18	152/158	2.2	6.78	–
21‐1	Puntarenas	6.8	Male	–	p.N1270S/p.M645R	Presymptomatic	+	8	–	5.04	7 IU/L	–	–	143/208	0.7	–	–
22‐1	San José	6.5	Female	–	p.N1270S/p.N1270S	Presymptomatic	–	8	–	3.52	0.03 g/L	–	1.09	100/197	1.1	–	–
23‐1	San José	9.9	Male	–	p.N1270S/p.N1270S	Presymptomatic	–	7	–	2.86	0.00 g/L	–	1.10	262/231	0.8	–	–
24‐1*	Cartago	11.8	Male	–	p.N1270S/p.N1270S	Hepatic	–	7	–	–	0.03 g/L	+	3.90	230/100	9.0	–	–
25‐1*	San José	7.3	Male	–	p.N1270S/–	Hepatic	+	6	–	8.26	0.02 g/L	–	–	130/54	2.0	3.77	–
26‐1*	San José	11.4	Male	–	p.N1270S/p.N1270S	Hepatic	–	8	–	21.07	0.00 g/L	–	3.10	191/86	2.4	–	–
27‐1	Alajuela	12.3	Male	–	–/–	Presymptomatic	–	5	–	9.46	0.19 g/L	–	1.10	25/12	1.4	13.7	–
28‐1*	San José	11.6	Male	–	p.N1270S/–	Hepatic	–	4	–	35.43	0.00 g/L	–	1.76	177/169	1.7	–	–

Abbreviations: ALF, acute liver failure; KF, Kayser–Fleischer; ULN, upper limit of normal; * = index case; ± = liver transplant; − = no; + = yes; † deceased.

**Figure 1:**
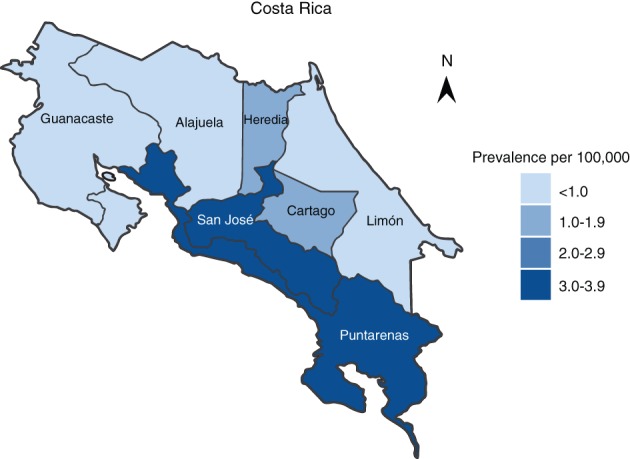
Prevalence of pediatric patients with Wilson Disease in Costa Rica by province

We estimated pediatric prevalence as the proportion of affected individuals over the population of individuals <18 years of age per region, according to the Costa Rican National Population Census data.^20^ The national prevalence of pediatric WD was estimated to be 2.2:100000. The proportion of affected individuals varied markedly depending on the region of the country from 0.6 to 3.9:100000 (Figure 1). The central province of San José was the most affected with a prevalence of 3.9:100 000, followed by the province of Puntarenas with 3.6:100 000. The difference in prevalence with previous adult population studies may be attributable to the natural history of the condition and its onset later in adulthood.^6^

Biallelic pathogenic variants were identified in 27 of the 34 individuals who met Leipzig criteria for WD. The most frequent pathogenic variant detected in our population in *ATP7B* (hg19) was NM_000053:c.3809A>G, p.Asn1270Ser (Table 2). The majority of pediatric WD cases (58.8%) were homozygous for this missense variant. Five different pathogenic variants were detected in WD cases: p.Asn1270Ser, p.Met645Arg, p.Leu708Pro, p.Thr1434Met, and p.Met665Ile. Individuals with unidentified variants had four of the 21 exons sequenced. Efforts are underway to identify variants in individuals with WD and ≤1 variant identified. From 106 individuals without WD, 23 were heterozygous for one pathogenic variant in *ATP7B* (19 p.Asn1270Ser, 2 p.Met645Arg, 1 p.Thr1434Met and 1 p.Met665Ile).

**Table 2:** Genotype frequency in pediatric patients with Wilson disease in Costa Rica

Genotype	Number of patients (n = 34)
*Homozygous*	
p.N1270S/p.N1270S	20 (58.8%)
*Compound heterozygous*	
p.N1270S/p.M645R	2 (5.9%)
p.N1270S/p.L708P	4 (11.7%)
p.N1270S/p.T1434M	1 (3.0%)
*One variant detected*	
p.N1270S/−	4 (11.7%)
p.M645R/−	2 (5.9%)
*No variants detected*	
−/−	1 (3.0%)

We documented the phenotype at onset with biochemical parameters. The most frequent clinical presentation was hepatic in 50% of cases (Table 3). The hepatic phenotype included: acute hepatitis, chronic hepatitis, hepatomegaly, or ALF. No patients presented as neuropsychiatric WD. All patients had decreased ceruloplasmin, most cases (32 of 34) in levels considerably below 50% of the lower normal limit (defined as 0.1 g/L or 28 IU/L for ceruloplasmin ferroxidase activity). Average urinary copper was 18.9 ± 30.7 μmol/ 24 hours, including patients under penicillamine challenge. The international scoring system for WD (EASL or Leipzig criteria) previously validated in pediatric patients, was highly sensitive capturing all patients in this cohort.^5^, ^21^

**Table 3:** Phenotypic presentations at diagnosis in pediatric patients with Wilson disease in Costa Rica

Phenotype	Number of patients (n = 34)	%	Jimenez et al. (n = 35)^a^^,^ b	%
Hepatic	11	32%	13	37%
Acute liver failure (deceased)	6 (1)	18%	11 (6)	31%
Presymptomatic	16	47%	6	17%
Coombs negative hemolytic anemia	1	3%	4	11%
Neurologic	0	0%	1	3%

a Jiménez et al.^19^

b Ten patients between the two studies (six with presymptomatic and four with hepatic phenotype) overlap due to a four‐year study intersection (2002‐2006).

**Figure 2:**
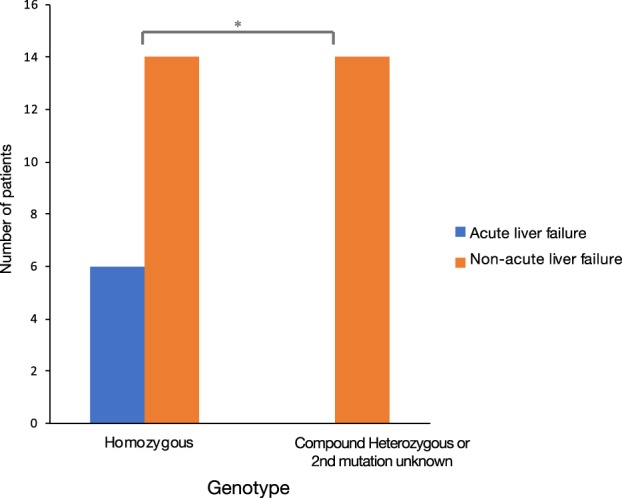
Genotype‐phenotype correlation. Comparison of homozygous p.Asn1270Ser phenotypes (n = 20) to heterozygous phenotypes (n = 14) in pediatric patients with Wilson disease (Chi‐squared, *P* < .05)

ALF occurred mainly in the context of chronic liver disease. Nonetheless, acute presentation was distinguished from chronic disease by symptom duration before diagnosis, clinical signs of chronic liver disease or portal hypertension. Most cases of ALF (4 of 6) were in more advanced stages of encephalopathy at time of presentation. Of the six cases presenting with ALF, four were females and two were male patients. Two patients with ALF were transplanted, three were not transplanted and survived, and one male patient died at age 9 years and 4 months due to fulminant liver failure (Tables 1 and 3).^22^ In the pediatric population with WD, all individuals with acute liver failure (n = 6) were homozygous for the p.Asn1270Ser variant (Figure 2). We found no significant difference in age of presentation between homozygous and heterozygous phenotypes. Sixteen of the 34 patients (47%) were detected in a presymptomatic stage through molecular cascade screening due to family history.

Jiménez et al.,^19^ characterized the pediatric population with WD at the same center before the advent of molecular testing, between 1992 and 2006 (Table 3). We documented a total of 34 patients diagnosed in a time span of 13 years and previously Jimenez et al. had documented a total of 35 cases diagnosed in a span of 14 years.^19^ There is a similar number of cases diagnosed per year between the two studies and a reduction in age of diagnosis from 10 ± 2 to 8.8 ± 3.4 years. Jimenez et al. documented six presymptomatic cases, captured due to family history of WD. We observed a total of sixteen presymptomatic cases. Although six presymptomatic patients from our cohort overlap with Jimenez et al. due to prior clinical diagnosis between 2002 and 2006, there is an increase of at least 30% of patients being captured presymptomatically.^19^ Mortality decreased from six cases to one case between the two studies.^19^

## 4 DISCUSSION

In this study, we describe what we have learned about the genotype and phenotype of the Costa Rican pediatric population with WD. Since our Institution serves as the national reference center for Clinical Genetics, Pediatric Gastroenterology, and Hepatology, we speculate to have captured nearly all pediatric WD cases in the nation. We compared data with a previous report from our center to demonstrate the impact of strategic measures in the delivery of genetics services.

First, we observed an increase in presymptomatic detection attributable to familial cascade genetic screening.^23^ Expanded cascade screening identified 16 presymptomatic patients with family history of WD. Earlier detection was possible with molecular testing for *ATP7B* genetic variants. We hypothesize early diagnosis and treatment prevented complications from chronic disease. In some instances, testing may have spared individuals from serial clinical evaluations or invasive testing. Since individuals with pathogenic variants in one allele can have slightly altered biochemical parameters, molecular testing may be helpful to distinguish between affected and carrier status. Future efforts to include the adult population could contribute to further characterize of the Costa Rican WD population.

Second, the number of pediatric cases identified over time was similar between Jimenez et al. (2009) and our cohort. Interestingly, a higher prevalence of cases in the central province of San José is consistent with prior reports and cases described here are moderately genetically homogenous.^6^, ^24^ Explanations for the higher incidence of WD in our population combined with a higher central region prevalence and a more genetically homogenous population, include consanguinity and a possible founder effect.^11^ Interestingly, the first reports of the p.Asn1270SSer variant were documented in the Mediterranean region (Sicily and Turkey) and later in Costa Rica.^11^, ^12^, ^25^ This missense variant has since been identified in various populations across the globe, specifically in Latino, Asian, African, and European populations.^26^ Rare recessive conditions, like WD, tend to be enriched in smaller, more genetically isolated populations.^27^ Founder effects are somewhat common among humans and have been identified before in Costa Rica.^7^, ^27^ However, the haplotype from a single common ancestor must be identified to determine if there is a founder effect for WD.^11^

Third, we observed that all pediatric cases with ALF had homozygous p.Asn1270Ser pathogenic variants. We find this observation noteworthy since the p.Asn1270Ser missense variant is located in the ATP hinge domain, which may be essential for protein function. Previous functional studies in *Saccharomyces cerevisiae* documented that wild type and other missense variants rescue delta ccc2, the yeast homologue of ATP7B,^8^ yet, the p.Asn1270Ser mutant could not rescue functionality of delta ccc2.^8^ Therefore, we hypothesize that the p.Asn1270Ser variant may have a highly deleterious effect, however, mutation status has not been demonstrated to relate to more severe phenotypes.^28^, ^29^

As genetic services in Costa Rica continue to grow, genetic knowledge continues to expand, and clinical applications increase, strategies to deliver services can be optimized for our population. Delivery of clinical services in the future may include widespread molecular screening in regions with high prevalence and copy number variant detection to improve sensitivity. Genetic sequencing offers the potential to be diagnostic, especially in cases where biochemical parameter interpretation is challenging. Molecular testing for more common recessive conditions in our nation has contributed to population characterization and earlier detection. We hope that our ongoing efforts lead to optimized molecular and biochemical screening for WD in Costa Rica.

## CONFLICT OF INTEREST

The authors have no conflicts of interest to declare.

## ETHICS APPROVAL AND INFORMED CONSENT

This investigation was approved by the Scientific Ethical Committee at the National Children's Hospital of Costa Rica (CEC‐HNN‐019‐2017) and followed Good Clinical Practice standards as well as local and international human research regulations. Data was de‐identified for analyses. The provision of individual consent forms is not required for review of medical records. The CENDEISSS Bioethics Committee approved the publication of this study.

## ANIMAL RIGHTS

This article does not contain studies with animal subjects performed by any of the authors.
